# Selected genetic aspects in the pathogenesis of the thyroid diseases

**DOI:** 10.1186/1756-6614-8-S1-A21

**Published:** 2015-06-22

**Authors:** Katarzyna Łącka

**Affiliations:** 1 Department of Endocrinology, Metabolism and Internal Medicine, University of Medical Sciences, Poznan, Poland

## 

According to genetic ethiopathogenesis, thyroid diseases may be divided into monogenic and polygenic (multifactorial) thyroid diseases.

Monogenic thyroid diseases are the following:

1. congenital defects of thyroid hormones biosynthesis as a result of dyshormonogenetic (majority goitrous) primary congenital hypothyroidism;

2. genetic familial defects of thyroid underdevelopment, including thyroid agenesis, thyroid hypoplasia and ectopia;

3. congenital protein-binding defects, including thyroxine-binding globulin (TBG) defects, albumin and prealbumin defects;

4. congenital resistance to thyroid hormone syndrome;

5. genetic non-autoimmune hyperthyroidism;

6. medullary thyroid carcinoma (MTC) (familial type of the MTC, and multiple endocrine neoplasia MEN 2 A and B), caused by *RET* gene mutations and inherited as the autosomal dominant mode.

About 10-15% of all cases of primary congenital hypothyroidism (CH) are associated with either a goitre or a normal thyroid gland. This type of CH has been linked to a number of defects in thyroid hormonogenesis and is caused by mutations in the following thyroid-specific genes: *hNIS, Tg, TPO, PDS, THOX2, DEHAL*. It is inherited by the autosomal recessive mode.

Only about 2% of primary congenital hypothyroidism cases (CH) due to the defects of the thyroid underdevelopment are familial with genetic background. This type of CH is associated with mutations in genes responsible for the growth or development of thyroid follicle cells such as *TSHR, TTF1, TTF2, PAX 8, NKX2,* or *Hoxa3*. Except for defect caused by a mutation of the TSHR gene, such inborn thyroid dysgenesis is associated with an increased incidence of birth defects (such as: respiratory distress, ataxia, muscle hypotomy, choreoathetosis, cleft palate, bilateral choanal atresia, hypoplastic epiglottis, spiky hair).

In addition, mutations in the *GNAS 1* gene may give congenital hypothyroidism associated with hypogonadisms and Albright’s osteodystrophy, which is the part of pseudohypoparathyroidism type IA and II (pseudopseudohypoparathyroidism).

The next group of monogenetic thyroid diseases with less or more evident thyroid dysfunction form the following:

1. TBG partial or complete deficiency and TBG excess due to *TBG* gene defects with an X-linked inheritance pattern;

2. Generalized, peripheral and pituitary resistance to thyroid hormones mainly caused by TRbeta gene mutations, inherited by the autosomal recessive.

The second group of thyroid diseases are the multifactorial (polygenic) ones. Among them the most frequent are:

1. Graves’ hyperthyroidism;

2. Autoimmune thyroiditis;

3. Thyroid carcinoma derived from thyroid follicle cells such as papillary and follicular thyroid carcinoma.

Although autoimmune thyroid diseases (AITD), including autoimmune thyroiditis and Graves’ hyperthyroidism with thyroid ophthalmopathy (TAO), are still unclear, they are defined as polygenic diseases resulting from genetic and environmental factors. The environmental factors which play a role in the development of AITD include iodine excess (with amiodarone treatment), selenium deficiency, stress, interferon alpha, bacterial and viral infections and, in the case of Graves’ hyperthyroidism, and tobacco smoking.

There are some genes whose polymorphisms have been confirmed in the development of and susceptibility to AITD. These are the following: 1. thyroid-specific genes such as the *Tg* gene and 2. genes modulating the immune system, such as HLA antigens, *CTLA4, PTPN22*, genes encoding proinflammatory cytokines, such as: IFN, TGF beta, IL 6, IL 4, IL1 beta, IL10, and others.

On the other hand, among the many genes involved in the development of differentiated thyroid carcinoma derived from thyroid follicle thyroid cells (papillary and follicular one), *BRAF* and *RET-PTC* seem to be the most important.

